# The Time Course of Event-Related Brain Potentials in Athletes’ Mental Rotation With Different Spatial Transformations

**DOI:** 10.3389/fnbeh.2021.675446

**Published:** 2021-06-15

**Authors:** Tian Feng, Yawei Li

**Affiliations:** ^1^Department of Social Sports, Physical Education College of Zhengzhou University, Zhengzhou, China; ^2^Department of Sports, Physical Education College of Zhengzhou University, Zhengzhou, China

**Keywords:** embodied cognition, egocentric rotation, object-based rotation, sport expertise, event-related potentials (ERP), mental rotation (MR)

## Abstract

Studies have found that athletes outperformed non-athletes in mental rotation tasks with both object-based and egocentric transformations (ET), but the effect of sport expertise on the processing stages (i.e., perceptual stage, rotation stage, and decision stage) remains conflicted. Bearing the view that the stages occur sequentially and the high temporal resolution of event-related brain potentials, this study focused on brain processing during mental rotation and was designed to determine the time course of electrophysiological changes in athletes and non-athletes. A total of 42 divers and non-athletes were recruited for the study. A mental body rotation task with object-based and egocentric transformation conditions was conducted, and the reaction time (RT), accuracy, performance stages, N2 latency, amplitude, and the amplitude of rotation-related negativity (RRN) were recorded. Behavioral results demonstrated higher accuracy for athletes at 120° and 180°. Moreover, as compared to non-athletes, the enlarged amplitude of N2 and RRN were confirmed in both transformations for athletes and were correlated with the performance stages and athletes’ professional training years. The present study provided a deeper insight into the relationship between sports training, behavior performance, and brain activity.

## Introduction

In daily life, imaging the spatial orientation in our mind can help us perform the actual action (i.e., turning the body left) quickly and accurately. The process by which an individual maintains and manipulates objects in space is called mental rotation (Shepard and Metzler, 1971). In the context of sports, most events require athletes to perceive, encode, and transform spatial information. The somersaults in gymnastics or diving, or the body turning in basketball and football are all performed by rotating the body around certain axes, so that rotation is one of the most crucial motor skills. From the perspective of embodied cognition, researchers believe that the learning of motor skills can enhance an individual’s cognitive abilities such as mental rotation. Confirmed by studies, a superior ability of mental rotation was found among athletes as compared to non-athletes (Moreau et al., 2015; Schmidt et al., 2015) and there was a significant positive correlation between mental rotation ability and sports expertise (Moreau et al., 2011). This superiority was attributed to the fact that athletes were able to automatically connect their nervous system with sensory information in a mental rotation task and exhibited an advantage that was consistent with kinematic characteristics and physical limitations (Sekiyama, 1982; Parsons, 1987; Jeannerod, 1994; Bonda et al., 1995; Kosslyn et al., 1998; Sauner et al., 2006; Thayer and Johnson, 2006). Specifically, studies found that for the poses that are possible for the human body to perform, the individual’s reaction time (RT) of mental rotation task was shorter (Amorim et al., 2006), proving a “spatial embodied” process. According to Wilson (2002) and previous studies of mental rotation (Feng et al., 2019), the embodied effect of mental rotation refers to the experience of physical activity that promotes the individual’s mental rotation and other spatial representation capabilities. In sports, the advantage of an athlete’s mental rotation is summarized as a spatial embodied effect; that is, when the space of mental rotation task matches with the physical action experience, task performance will be promoted and expert advantage will be found (Feng et al., 2017, 2019).

According to the reference frame of rotation, mental rotation is divided into two types: object-based representation and egocentric transformation (Zacks et al., 2002). In the former, subjects perform rotation operations in the third-person perspective, such as watching the leftward movement demonstrated by a physical education teacher. In the latter, subjects rotate themselves from a first-person perspective, such as imagining themselves making a leftward turn. Most studies have confirmed that athletes outperform non-athletes in egocentric transformation (Steggemann et al., 2011; Kaltner and Jansen, 2015; Heppe et al., 2016), Kaltner et al. (2014) and Kaltner and Jansen (2016) used human images for object-based and egocentric transformation and found that the advantage for athletes with rotation experience (i.e., somersaults) only exists in egocentric transformation. However, this does not seem to be the case for object-based transformation. Some studies reported the advantage for athletes in object-based transformation (Sylvie et al., 2002, 2004; Moreau et al., 2011, 2012; Pietsch and Jansen, 2012; Kaltner and Jansen, 2015; Schmidt et al., 2015), while some studies failed to find supportive results (Steggemann et al., 2011; Pasand et al., 2015; Schmidt et al., 2015). This conflict may be related to the stimulus type of the experimental task. Studies using stimulus images without movement information, ranging from letters (Pasand et al., 2015) to abstract graphics (Schmidt et al., 2015) may find it hard to yield similar results or group differences. Thus, the present study aims to use the image of the human body to elicit object-based representation and egocentric transformation.

According to the phases of information processing, mental rotation is subdivided into the perceptual stage, rotation stage, and decision stage (Shepard and Cooper, 1986; Corballis, 1988; Heil and Rolke, 2002). Specifically, the performance in the perceptual and decision stages is the RT when the stimulus is not rotated, and the mental rotation speed represents the performance of the rotation stage. Previous studies have found that athletes present different stage advantages in mental rotation with different spatial representations. In object-based transformation, athletes outperformed non-athletes only in the perceptual and decision stages. In egocentric transformation, athletes were better at the perceptual, decision, and rotation stages (Feng et al., 2017).

Importantly, every stage of mental rotation occurs sequentially, and event-related potentials (ERP) can accurately reveal the time course of brain processing. However, few studies have noted the importance of brain processing among individuals with different sports experiences during mental rotation. As per the neurophysiological evidence of mental rotation, the parietal region’s rotation-related negativity (RRN) is a classical neurophysiological indicator. RRN occurs between 300 and 800 ms after the onset of stimuli and is dependent on the angular disparity (Heil and Rolke, 2002; Horst et al., 2012). Previous studies such as that of Yin (2015) used letters and hand images as material to compare the brain characteristics of athletes and non-athletes and found that athletes had larger amplitudes of RNN in egocentric transformation, but found no group difference in object-based transformation. In contrast, Song (2008) investigated the RRN of martial arts athletes and non-athletes during the mental rotation of letters and confirmed larger RRN amplitudes for the athlete. Moreover, two problems exist in the above studies. First, their task stimulus is mostly letters or a certain part of the body (hand), which ignores Heinen’s claim of systemic and specific task stimulus (Heinen, 2013). Therefore, they may not be able to accurately demonstrate the traits of brain activity in athletes’ mental rotation caused by sport expertise. Secondly, only focusing on RRN, a relatively late component of mental rotation, may not be enough for investigating the ability of mental rotation. Lyu’s research found that the early anterior N2 may reflect the individual’s perceptual ability of stimuli (Lyu et al., 2017), but whether there is some relationship between N2 and perceptual and decision stages of mental rotation remains unclear. Therefore, the present study sets out to explore the relationship between the characteristics of behavior results and brain processing in mental rotation among athletes and non-athletes.

Based on the stage advantage of athletes’ mental rotation with body stimuli in different transformations, the present study aims to explore the time course of brain processing in mental rotation for individuals with various sports expertise, and the relationship between behavioral performance and ERP results. It was hypothesized that: (1) Athletes will show better behavioral performance than non-athletes; (2) Athletes will perform shorter N2 latency and larger peak amplitudes of N2, and that the latter will be related to their performance in the perceptual and decision stages; and (3) The RRN amplitude for athletes will be larger than the RRN amplitude for non-athletes and related to the athletes’ performance in the rotation stage.

## Materials and Methods

### Participants

Forty-two participants, including 20 athletes (10 men and 10 women) and 22 non-athletes (12 men and 10 women), participated in the experiment. There was no significant difference in age between the two groups (athletes: aged 20.00 ± 3.50 years, non-athletes: aged 21.11 ± 2.94 years, *t* = −1.054, *p* = 0.299). The athletes group was comprised of divers from the Beijing diving team. The athletes’ training age was between 5 and 13 years, and they practiced for about 36 h per week. The non-athletes group was comprised of students recruited from the Shanghai University of Sport, who had never participated in professional sports training and did not have regular physical activities. This study was approved by the Ethics Committee of the Shanghai University of Sport (2017036), and informed consent was obtained from the participants and their parents prior to participation, wherein they also received gifts or cash rewards after completion of the experiment.

### Materials

**Figure 1:**
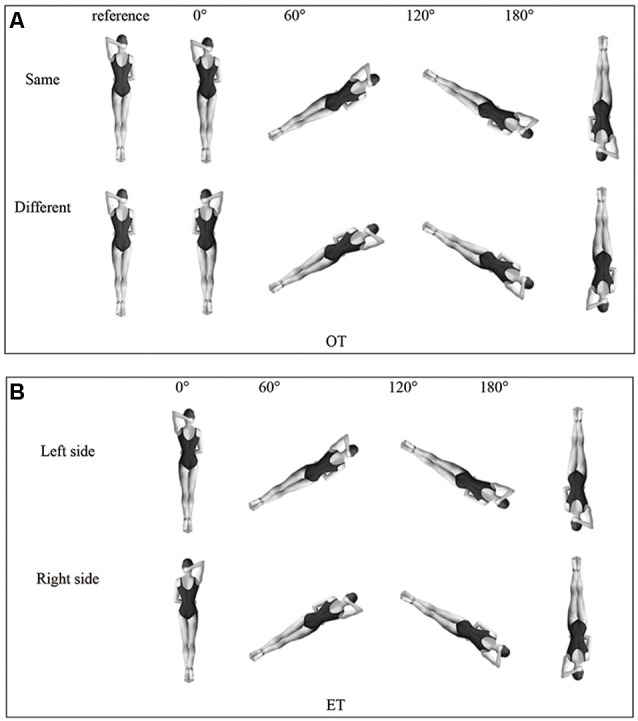
Task stimuli in OT condition **(A)** and ET condition **(B)**. Copyright by QA International, 2017. All rights reserved. Abbreviations: OT, object-based transformations; ET, egocentric transformations.

The experiment included the mental rotation task of the object-based transformations (OT) condition and the egocentric transformations (ET) condition, and the image for experimental stimulus was the back of a woman wearing a dark swimsuit with the elbow of one arm on the head and the other arm placed on the waist (see Figure 1). In the OT condition, one image at a certain angle was presented; in the ET condition, two images were presented. The left image was an upright target and the right image was a rotated identical (same) or mirror-reversed (different) image of the target (Figure 1A). The stimuli were presented at a size of 4 × 4 cm in black and white. Considering the repetition time of the ERP experiment and the setting of angles in previous studies (Wijers et al., 1989), the task was conducted at four angles, including 0°, 60°, 120°, and 180° (Figure 1B). The task was designed and displayed with E-Prime 2.0 (Psychology Software Tools, Sharpsburg, Pennsylvania) on three ThinkPad laptops with 14-inch screens.

### Procedure

The experiment was conducted in a quiet room by two experimenters. First, the participants washed and dried their hair before they completed a questionnaire with individual information. They were then seated in front of a screen at a distance of 60 cm and wore an electrode cap. After reading the task instructions, participants were asked to practice 20 trials with feedback. The accuracy had to be higher than 80%, that is, wrong answers were supposed to occur in fewer than four trials, after which the formal experiment could start. Otherwise, participants needed to perform an additional 20 trials to meet the requirements. In the Object-based transformations (OT) condition, the participants were asked to determine whether the two images were the same as quickly and accurately as possible. In the Egocentric transformations (ET) condition, the participants needed to decide which arm the woman had bent above her head in the picture. The “F” key represented left/same and the “J” key represented right/different. The experiment consisted of 2 (transformation types) × 4 (angular disparity) × 2 (same and different/left and right) × 30 (repetition) trials for a total of 480 trials, which were randomly divided into four blocks. In each trial, the screen showed a fixation point (1,000 ms−1,500 ms), and then the image/s was/were presented until the “F” or “J” keys were pressed by the participant. Responses submitted more than 3,000 ms after the presentation of the stimulus were considered errors. The next trial started after a blank screen of 1,000 ms. The whole experiment lasted about 80 min.

### EEG Data Recording and Analysis

EEG signals were recorded with a 64-channel EEG EasyCap connected to the BrainAmp MR Plus signal amplifier (Brain Products GmbH, Munich, Germany). The signals were filtered online with 100 Hz and sampled at 1,000 Hz. Horizontal and vertical electrooculograms were monitored for detecting eye movements and blinks. The impedance of electrode points was kept less than 5 kΩ. The raw EEG signal was pre-processed offline with the BrainVision Analyzer (v2.0, Brain Products GmbH, Munich, Germany). First, the reference electrodes were re-referenced to TP9 and TP10 (left and right mastoids), after which the data were filtered using a 0.5−30 Hz band-pass filter. An EEG amplitude exceeding ± 200 μV or a gradient value more than 50 μV/ms was excluded by semi-automatic detection. Eye electricity was corrected by independent component analysis. After segmenting the data, the baseline correction (from −200 ms to 0 ms before the onset of the stimulus) was applied. The data of 60 repetitions for every two transformation types and four angles were then averaged. The segmentation time in the OT condition ranged from 200 ms prior to the stimulus to 1,500 ms after the stimulus, and the segmentation time in the ET condition was from −200 ms to 800 ms. Finally, the N2 and Rotation-related negativity (RRN) components were obtained. According to previous studies, anterior N2 and parietal RRN are crucial indicators of mental rotation ability. The present study analyzed the electrode F3, Fz, and F4 of N2 and the electrode P3, Pz, and P4 of RRN. The latency and peak amplitude of N2 were measured at the peak of 210−290 ms, and the average RRN amplitude in the OT and ET conditions was calculated as the mean amplitude within 550–750 ms and 400–600 ms, respectively.

### Statistical Analysis

Participants who had more than three SDs above the mean of Reaction time (RT), rotation speed, Event-related potentials (ERP) peak, and average amplitude, and accuracy lower than 85% (one athlete and two non-athletes), were excluded. According to Just and Carpenter (1985), the RT when stimulus material was not rotated (that is, 0°) was taken as the performance of the perceptual and decision stages. The rotation speed was used as the performance of the rotation stage and it was the average of the ratio of the angle at each angle to the RT. The calculation formula is: rotation speed = $\left(\frac{60}{RT_{60^{\circ }}}+\frac{120}{RT_{120^{\circ }}}+\frac{180}{RT_{180^{\circ }}}\right)÷3\times$ 1,000, where the unit is represented in degree per second (°/s). The calculation above was only carried out with the correct trials. After transforming the RTs of the two groups in two conditions into a logarithmic base (ln) and conducting arcsine conversion for the accuracy and stage performance, analysis of variance (ANOVA) assumptions on normality and the homogeneity of variances were verified for each sample (Kolmogorov-Smirnov and Levene’s tests were non-significant in all cases). Repeated-measures analysis of variance (R-M ANOVA) for RT and accuracy were calculated with the between-subject factor of both groups (athletes and non-athletes) and the within-subject factors of transformation type (OT, ET) and angular disparity (0°, 60°, 120°, and 180°). For the performance stage, R-M ANOVAs for the RT at 0° and rotation speed were conducted with the between-subject factor of the groups (athletes and non-athletes) and the within-subject factor of transformation type (OT, ET). Similarly, the ERP data were analyzed by R-M ANOVA, with the latency and peak amplitude of N2 and the average amplitude of RNN as dependent variables, the group (athletes and non-athletes) as between-subjects variables, and the transformation type (OT, ET), angular disparity (0°, 60°, 120°, and 180°), and electrode (F3, Fz, and F4/P3, Pz and P4) as between-subject variables. Bonferroni tests were used for *post hoc* tests for main effects and interactions. Due to the well-known gender differences in MR, we tested the effect of gender on the performance of athletes and non-athletes but found no significant effect of gender in both kinds of transformations (all *p* > 0.526, *d* < 0.29). Given this result, gender was not included as a factor during all the analyses.

## Results

### Behavioral Results

#### Reaction Time

The ANOVA revealed significant main effects of transformation type, *F*_(1,37)_ = 347.978, *p* < 0.001, $\eta_{\text{p}}^{2}$ = 0.909, and angle disparity, *F*_(3,111)_ = 126.229, *p* < 0.001, $\eta_{\text{p}}^{2}$ = 0.783, but not of the group, *F*_(1,37)_ = 0.011, *p* = 0.919, $\eta_{\text{p}}^{2}$ = 0.000, showing that no difference exists in RTs between athletes (OT: 1127 ± 159 ms, ET: 643 ± 108 ms) and non-athletes (OT: 1197 ± 339 ms, ET: 643 ± 208 ms). Additionally, there was a significant interaction between transformation type and angle disparity, *F*_(3,111)_ = 38.034, *p* < 0.001, $\eta_{\text{p}}^{2}$ = 0.521. *Post hoc* tests found that the RT at every angle was different in the OT and ET conditions, except for 0° and 60° (Table 1). The RT was faster in the ET condition than that in the OT condition (all *p* < 0.001, *d* > 10.67). Other interactions did not reach significance (*F* < 1.097, *p* > 0.302, $\eta_{\text{p}}^{2}$ < 0.030).

**Table 1:** Reaction time per group in object-based transformations (OT) and egocentric transformations (ET) conditions (*M* ± *SE*).

OT	Angle (°)	0	60	120	180
	RT (ms)	883 ± 33	1041 ± 36	1285 ± 50	1564 ± 73
	0	−	(*d* = 4.58)***	(*d* = 9.48)***	(*d* = 12.02)***
	60		−	(*d* = 5.60)***	(*d* = 9.08)***
	120			−	(*d* = 4.46)***
	180				−
ET	Angle (°)	0	60	120	180
	RT (ms)	552 ± 19	561 ± 21	656 ± 27	865 ± 50
	0	−	(*d* = 0.45)^n.s.^	(*d* = 4.45)***	(*d* = 8.28)***
	60		−	(*d* = 3.93)***	(*d* = 7.93)***
	120			−	(*d* = 5.20)***
	180				−

n.s, non-significant; *** *p* < 0.001.

#### Accuracy

**Figure 2:**
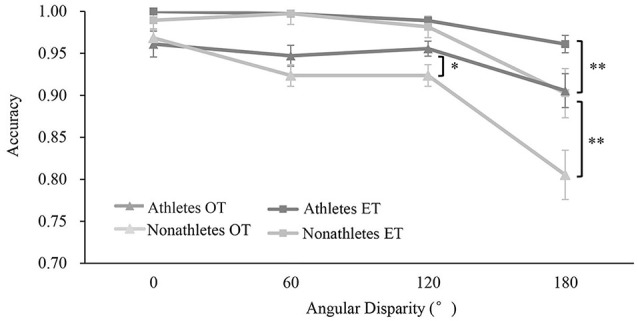
Accuracy (M ± SE) of athletes and nonathletes with different transformation types. *p < 0.05, **p < 0.01.

The ANOVA of accuracy demonstrated significant main effects of the transformation type, *F*_(1,37)_ = 53.042, *p* < 0.001, $\eta_{\text{p}}^{2}$ = 0.602, angle disparity, *F*_(3,111)_ = 27.501, *p* < 0.001, $\eta_{\text{p}}^{2}$ = 0.440, and group, *F*_(1,37)_ = 14.746, *p* < 0.001, $\eta_{\text{p}}^{2}$ = 0.296. Additionally, significant interaction between groups and angle disparity was revealed, *F*_(3,111)_ = 5.634, *p* < 0.01, $\eta_{\text{p}}^{2}$ = 0.139. *Post hoc* tests found that the accuracy of athletes at 120° in the OT condition (0.956 ± 0.038) and 180° in both conditions (OT: 0.906 ± 0.086, ET: 0.961 ± 0.044) were higher than those of non-athletes (120°: OT: 0.924 ± 0.056, 180°: OT: 0.805 ± 0.128, ET: 0.903 ± 0.114, all *p* < 0.05, *d* > 0.27, Figure 2). However, the accuracy of athletes (0°: OT: 0.961 ± 0.065, ET: 1.00 ± 0.000, 60°: OT: 0.947 ± 0.053, ET: 0.997 ± 0.012) and non-athletes (0°: OT: 0.968 ± 0.048, ET: 0.990 ± 0.046, 60°: OT: 0.944 ± 0.077, ET: 0.997 ± 0.011) at 0° and 60° in both conditions and 120° in the ET condition (athlete: 0.989 ± 0.021, non-athlete: 0.982 ± 0.030) were not significant (all *p* > 0.208, *d* < 0.12). Additionally, the accuracy of the non-athletes’ group in the OT and ET conditions at 120° was higher than that at 180° (all *p* < 0.01, *d* > 0.95), but not of those at 0° and 60° as well as all the angles of the athletes (all *p* > 0.311, *d* < 0.10). Other interactions were not significant (*F* < 2.241, *p* > 0.121, $\eta_{\text{p}}^{2}$ < 0.060).

#### Mental Rotation Stages

The ANOVA results for perceptual and decision stages showed significant main effects of the transformation type for 0° RT, *F*_(1,37)_ = 204.320, *p* < 0.001, $\eta_{\text{p}}^{2}$ = 0.854, but not of the group, *F*_(1,37)_ = 0.006, *p* = 0.940, $\eta_{\text{p}}^{2}$ = 0.000 and their interaction, *F*_(1,37)_ = 3.504, *p* = 0.070, $\eta_{\text{p}}^{2}$ = 0.091, which meant that the perceptual and decision time for ET was shorter than that of OT, but there was no significant difference in 0° RT between athletes (OT: 859 ± 115 ms, ET: 572 ± 100 ms) and non-athletes (OT: 906 ± 258 ms, ET: 532 ± 128 ms).

The ANOVA results for the rotation speed indicated significant main effects of the transformation type, *F*_(1,37)_ = 429.717, *p* < 0.001, $\eta_{\text{p}}^{2}$ = 0.925, with a faster rotation speed for ET. No significant main effects of group, *F*_(1,37)_ = 0.411, *p* = 0.526, $\eta_{\text{p}}^{2}$ = 0.012, or the interaction between transformation type and group were found, *F*_(1,37)_ = 0.986, *p* = 0.328, $\eta_{\text{p}}^{2}$ = 0.027. This result indicates that no significant differences existed in the rotation speed between athletes (OT: 93 ± 18°/s, ET: 171 ± 32°/s) and non-athletes (OT: 95 ± 28°/s, ET: 180 ± 37°/s).

### ERP Results

#### N2

The two groups (athletes and non-athletes) × two transformation types (OT, ET) × four angular disparities (0°, 60°, 120°, and 180°) × three electrode point positions (F3, Fz, and F4) RM ANOVA of N2 latency showed significant main effects of angle disparity wherein *F*_(1,111)_ = 3.350, *p* < 0.05, $\eta_{\text{p}}^{2}$ = 0.095 and the three-way interaction between transformation type, angle disparity, and group *F*_(1,111)_ = 6.248, *p* < 0.01, $\eta_{\text{p}}^{2}$ = 0.163. *Post hoc* tests found that the N2 latencies of non-athletes were shorter at the angle of 0° (241 ± 31 ms) than at 60° (254 ± 30 ms) and 120° (263 ± 29 ms) in the OT condition (all *p* < 0.05, *d* > 0.43), but not for 180° (253 ± 35 ms) and all the angles in the ET condition (all *p* > 0.15, *d* < 0.21). The N2 latency of athletes also showed a difference between 0° (238 ± 31 ms) and 60° (246 ± 22 ms), as well as 60° and 120° (269 ± 23 ms) in the ET condition, with the shortest latency of 0° (all *p* < 0.05, *d* > 0.30), though no significant difference was found between 180° (255 ± 33 ms) and other angles in the ET condition and all the angles in the OT condition (all *p* > 0.180, *d* < 0.12). Additionally, the results did not show any difference between the athletes and non-athletes at all angles in both conditions (all *p* > 0.443, *d* < 0.05). The main effects and interaction effects of other factors were not significant, wherein *F* < 1.256, *p* > 0.292, $\eta_{\text{p}}^{2}$ < 0.038.

**Figure 3:**
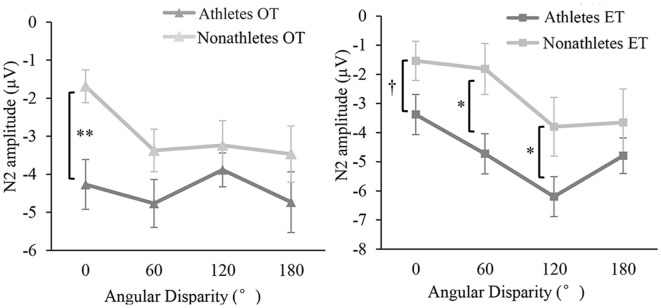
The peak amplitude of N2 as a function of the angle at Pz per group in OT and ET conditions (M ± SE). In the left panel, the dark gray line with the triangle denotes the athletes in the OT condition, and the light gray line with the triangle denotes non-athletes in the OT condition. In the right panel, the dark gray line with the square denotes the athletes in the ET condition, and the light gray line with the square denotes non-athletes in the ET condition. ^†^*p* < 0.1, *p < 0.05, **p < 0.01.

**Figure 4:**
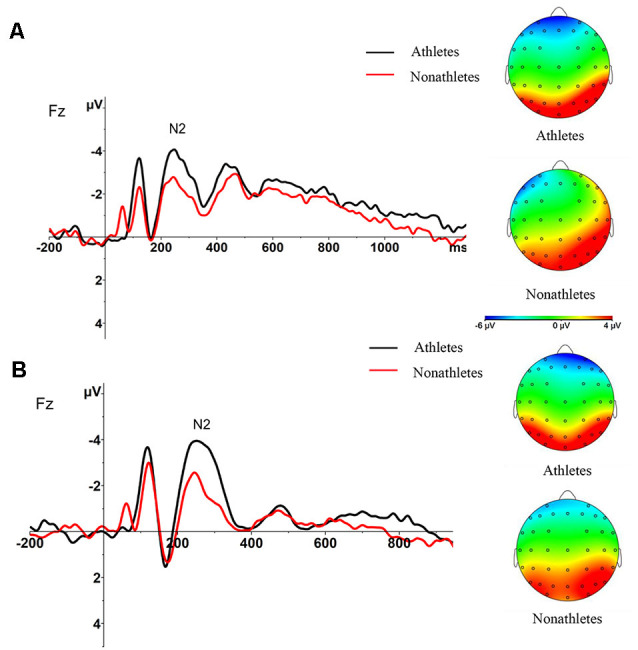
Peak amplitude of N2 and spatial distribution over all electrode at Fz in OT **(A)** and ET **(B)** condition per group (M ± SE).

The ANOVA of the N2 amplitude indicated significant main effects of group, *F*_(1,37)_ = 4.521, *p* < 0.05, $\eta_{\text{p}}^{2}$ = 0.131, angle *F*_(1,111)_ = 13.827, *p* < 0.001, $\eta_{\text{p}}^{2}$ = 0.315, and the interaction between transformation type and angle disparity *F*_(1,111)_ = 7.744, *p* < 0.001, $\eta_{\text{p}}^{2}$ = 0.205. The interaction between transformation type, angle disparity, and group was marginally significant, and *F*_(1,111)_ = 2.936, *p* = 0.051, $\eta_{\text{p}}^{2}$ = 0.089. *Post hoc* tests confirmed that the difference of N2 amplitude between athletes and non-athletes existed at 0° of the OT condition (average amplitude of every electrode in athletes: −4.27 ± 2.91 μV, non-athletes: −1.69 ± 2.08 μV) and at 0° (athletes: −3.40 ± 2.33 μV, non-athletes: −1.49 ± 2.60 μV), 60° (athletes: −4.73 ± 3.54 μV, non-athletes: −2.39 ± 4.09 μV), and 120° (athletes: −6.20 ± 3.52 μV, non-athletes: −4.50 ± 4.65 μV, all *p* < 0.065, *d* > 0.68) of the ET condition, which showed a larger mean amplitude for athletes (Figures 3, 4). Moreover, *post hoc* results did not show any angle or transformation difference within the athletes and non-athletes groups (all *p* > 0.115, *d* < 0.20). The main effects and interactions of other variables were not significant, wherein all *F* < 1.882, *p* > 0.142, $\eta_{\text{p}}^{2}$ < 0.057.

#### RRN

**Figure 5:**
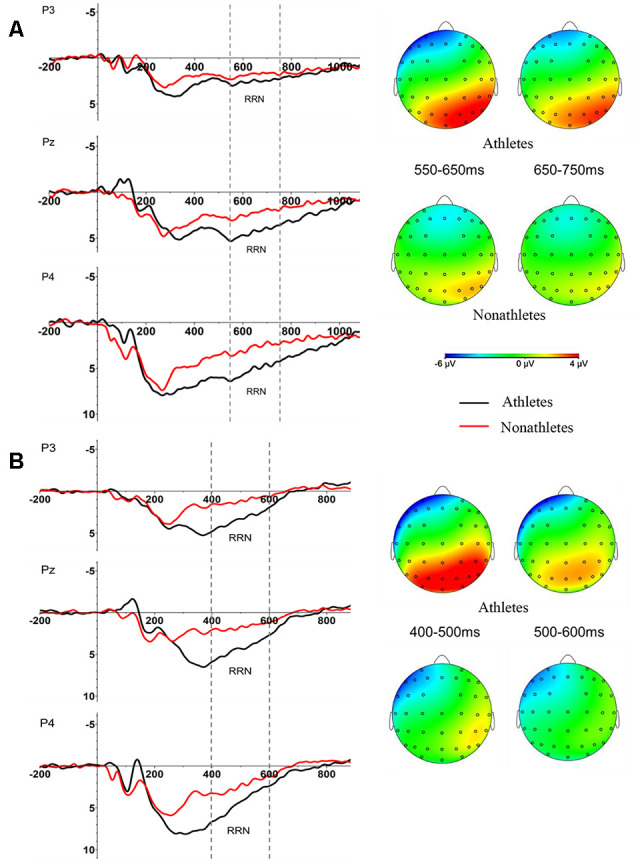
Average amplitude of rotation-related negativity (RRN) at P3, Pz and P4 and spatial distribution over all electrode in OT **(A)** and ET **(B)** condition per group (M ± SE).

**Figure 6:**
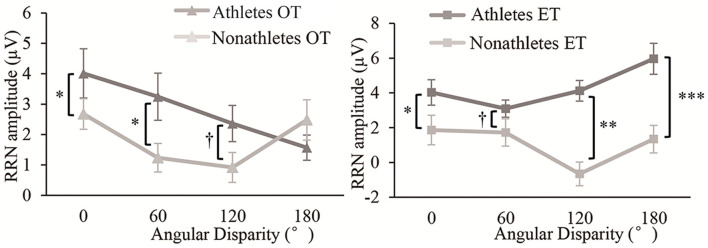
Average amplitude of RRN at Pz per group in OT and ET condition (M ± SE). ^†^*p* < 0.1, *p < 0.05, **p < 0.01, ***p < 0.001.

Based on the two groups (athletes and non-athletes) × two transformation types (OT, ET) × four angular disparities (0°, 60°, 120°, and 180°) × three electrode point positions (F3, Fz, and F4) RM Analysis of Variance (ANOVA) analysis of the average amplitude of RRN, we found significant main effects of the group, angle, and electrodes, and several significant interactions (Table 2). Moreover, the interactions between the transformation type, angle disparity, and group were significant. *Post hoc* tests found that in the OT condition, the differences of RRN amplitude between athletes and non-athletes existed at 0°, 60°, and 120° (all *p* < 0.094, *d* > 0.30) but not at 180° (*p* = 0.134, *d* = 0.19). Moreover, in the ET conditions, athletes’ RRN amplitudes were significantly larger than those of non-athletes at all angles (all *p* < 0.083, *d* > 0.34, Figures 5, 6). However, the transformation and angle effects were not significant within each group (all *p* > 0.260, *d* < 0.11). Besides, *post hoc* tests for the interactions between the transformation type, angle, and electrode found more amplified RRN at P4 than that at P3 and Pz (all *p* < 0.046, *d* > 0.40), but no electrode effect for each transformation or angle (all *p* > 0.337, *d* < 0.09).

**Table 2:** ANOVA results of average amplitude of rotation-related negativity (RRN).

Effects	*F* (df)	*p*	$\eta_{\text{p}}^{2}$
Group	7.062 (1, 37)	0.014	0.243
Transformation type	0.156 (1, 37)	0.697	0.007
Angle	9.822 (1, 111)	0.000	0.309
Electrode	11.213 (1, 74)	0.001	0.338
Group × transformation type	9.593 (1, 37)	0.005	0.304
Group × angle	0.978 (1, 111)	0.399	0.043
Group × electrode	0.690 (1, 74)	0.449	0.030
Transformation type × angle	4.502 (1, 111)	0.024	0.170
Transformation type × electrode	7.380 (1, 74)	0.009	0.251
Angle × electrode	22.128 (1, 222)	0.000	0.501
Transformation type × angle × group	5.355 (1, 111)	0.014	0.195
Transformation type × electrode × group	0.944 (1, 74)	0.341	0.043
Angle × electrode × group	0.532 (1, 222)	0.689	0.024
Transformation type × angle × electrode	13.704 (1, 222)	0.000	0.384
Transformation type × angle × electrode × group	1.454 (1, 222)	0.232	0.062

#### Correlation Analysis

**Figure 7:**
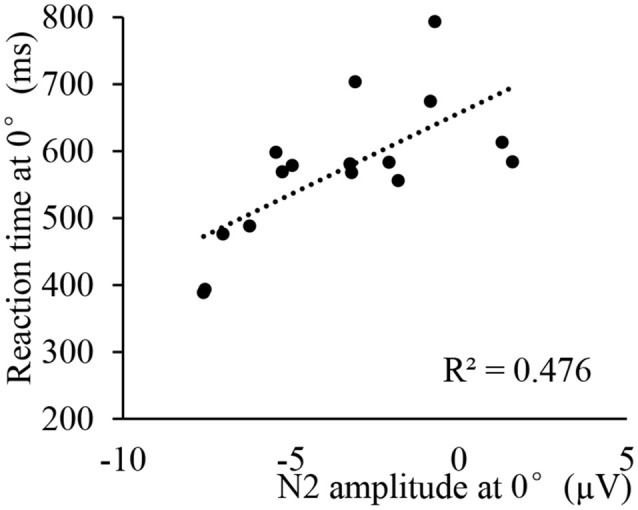
Correlation between N2 amplitude (Pz) and RT at 0° for athletes in ET condition.

**Figure 8:**
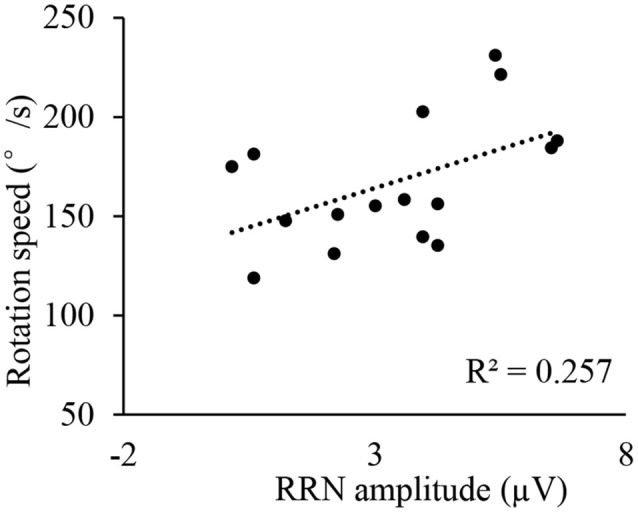
Correlation between RRN amplitude (P3) and rotation speed for athletes ET condition.

**Figure 9:**
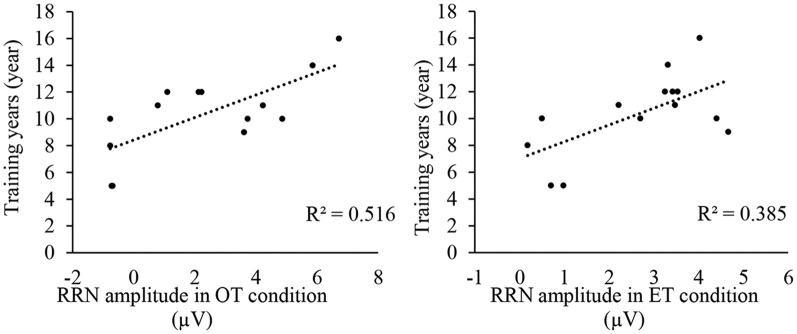
Correlation between RRN amplitude (Pz) and training years for athletes in each condition.

Previous studies have found that N2 represents the perception of stimuli (related to the perception and decision stages), and RRN represents the operation on the stimulus (related to the rotation stage). Therefore, we analyzed the correlation between the peak amplitude of N2 and the performance at the perception and decision stages (RT at 0°), as well as the average amplitude of RRN and the rotation stage (rotation speed), respectively. Results showed a significant positive correlation between the athletes’ N2 amplitudes at 0° and the RT at 0° in ET conditions (all *r* > 0.523, *p* < 0.05, Table 3 and Figure 7), and there was a significant positive correlation between the RRN amplitude at P3 and the rotation speed at 180° (*r* = 0.506, *p* < 0.05, Table 4 and Figure 8). Additionally, to examine the relationship between sport expertise and ERP, we also analyzed the correlation between the athletes’ professional training years, N2 amplitude, and RRN amplitude. A significant positive correlation between RRN amplitude and the athletes’ professional training years was demonstrated at three electrodes in each transformation type (all *r* > 0.621, *p* < 0.01, Tables s 5, 6 and Figure 9).

**Table 3:** Values of correlation coefficients between N2 amplitude and reaction time (RT) at 0° in OT and ET conditions.

Angle (°)	0			60			120			180		
Electrode	F3	Fz	F4	F3	Fz	F4	F3	Fz	F4	F3	Fz	F4
N2 in OT
RT at 0°	0.052	0.116	−0.133	0.201	0.111	−0.203	0.056	0.181	0.199	0.260	0.166	0.230
N2 in ET
RT at 0°	0.523*	0.601**	0.599*	0.221	0.197	−0.033	0.199	0.100	0.127	0.263	0.001	0.070

*p < 0.05, **p < 0.01.

**Table 4:** Values of correlation coefficients between RRN amplitude and rotation speed in OT and ET conditions.

Angle (°)	0			60			120			180		
Electrode	P3	Pz	P4	P3	Pz	P4	P3	Pz	P4	P3	Pz	P4
RRN in OT
Rotation speed	0.222	0.169	0.005	−0.016	0.209	0.174	0.097	−0.195	0.127	0.165	0.093	0.223
RRN in ET
Rotation speed	0.131	0.002	0.205	0.214	0.031	0.180	0.191	0.204	0.265	0.506*	0.211	0.256

*p < 0.05.

**Table 5:** Values of correlation coefficients between N2 amplitude and training years in OT and ET conditions.

Angle (°)	0			60			120			180		
Electrode	F3	Fz	F4	F3	Fz	F4	F3	Fz	F4	F3	Fz	F4
N2 in OT
Training years	−0.207	−0.141	−0.120	0.010	0.250	0.135	0.155	−0.217	−0.077	0.104	0.167	0.063
N2 in ET
Training years	0.201	−0.009	0.195	0.179	0.065	0.100	−0.318*	0.054	−0.182	−0.033	0.219	0.135

*p < 0.05.

**Table 6:** Values of correlation coefficients between RRN amplitude and training years in OT and ET conditions.

Angle (°)	0			60			120			180		
Electrode	P3	Pz	P4	P3	Pz	P4	P3	Pz	P4	P3	Pz	P4
RRN in OT
Training years	0.299	0.622**	0.626**	0.716**	0.692**	0.744***	0.297	0.666**	0.680**	0.860***	0.793***	0.623*
RRN in ET
Training years	0.195	0.062	0.705**	0.664**	0.301	0.621**	0.698**	0.704**	0.665*	0.679**	0.711***	0.696**

*p < 0.05, **p < 0.01, ***p < 0.001.

## Discussion

The present study examined the time course of mental rotation for athletes and non-athletes in object-based and egocentric transformation. In terms of behavioral results, we found that athletes had a higher accuracy rate than non-athletes, but there was no difference in performance in RT or each stage. Regarding the ERP results, it was shown that the athletes’ N2 amplitudes at 0° in OT conditions and 0°, 60°, and 120° in ET conditions were significantly larger than those of non-athletes and were related to the performance of the perception and decision stages (RT at 0°). In addition, the athletes’ RRN amplitudes were significantly larger than those of non-athletes. Moreover, the RRN amplitude was significantly correlated with the performance of the rotation stage (rotation speed) and the athletes’ professional training years.

The results showed that the accuracy of athletes was significantly higher than that of non-athletes in each condition (OT and ET), which corroborated Hypothesis 1. This result was consistent with Moreau (2012), who found that the accuracy of gymnasts and wrestlers was significantly higher than that of non-athletes. Besides, the accuracy of athletes was found to be higher at a larger angle (120° and 180°), which was supported by Steggemann et al. (2011) and Kaltner and Jansen (2015). They confirmed that sports experts had the advantage of egocentric mental rotation at unusual rotation angles (Habacha et al., 2017). This result confirms the hypothesis of functional equivalence of embodied cognition, which can be explained by the processing of movements. As a type of embodied spatial transformation, movement embodiment refers to the process of observation, imagination, and other representations consistent with the actual operations (Decety, 2002). All diving movements require head-down entry into the water, and some movements even start with a headstand, so watching or performing head-down movements was familiar for the diver. Therefore, athletes can excel when they perform a mental transformation on familiar movements, and even show a priming effect (Amorim et al., 2006). Concerning the object-based transformation and egocentric transformation, most studies found higher accuracy and faster RT in the ET condition than that in the OT condition (Jola and Mast, 2005; Jansen and Lehmann, 2013; Kaltner et al., 2014; Kaltner and Jansen, 2015), which is consistent with the present study. Embodied cognition states that human perception is functionally equivalent to their surrounding physical phenomena (Heinen, 2013). The effect of transformations could be explained by the idea of the heterogeneity of transformations. In daily life, we learn to rotate ourselves and other things from a first-person perspective. Therefore, a person can adopt more body-related experiences to accelerate judgments during an egocentric transformation.

Concerning ERP results, N2 is considered to be a cognitive and intrinsic ERP component. The earliest report of N2 was found in a visual oddball task, so that complex novel stimuli can cause a larger frontal N2 than a simple one can. Thus, N2 becomes the ERP component for detecting the novelty of stimuli (Courchesne et al., 1975; Folstein and Van Petten, 2008). The present study found that, in the ET condition, N2 amplitude in the frontal area increased with the angle increment from 0° to 120° for both groups, which revealed the angle effect of N2 amplitude. Supported by the research of Lyu et al. (2017), with the increase of the rotation angle, the stimulus and the upright image became more dissimilar, which made the N2 amplitude higher. At the same time, we found that N2 amplitudes at 180° did not continue to increase in the ET condition, but they were close to that at 120° in the non-athlete group or even smaller than that at 120° in the diver group. The reason may be attributed to the participants’ different strategies of judging when the stimulus was rotated upside down. We investigated a few subjects and found that when the stimulus was completely inverted, some subjects formed a judgment of “right is left, and left is right,” which reduces the novelty of the stimulus to a certain extent so that the N2 amplitude at this angle was close to 120° or less. Also, results showed that the angle effect of N2 amplitude was not clear in the OT condition. A possible explanation for this might be that the novelty of stimulation in the object-based transformation did not increase with the rotation angle. However, previous ERP research of mental rotation often used letters (Riečanský and Jagla, 2008; Núñez-Peña and Aznar-Casanova, 2009) or hands (Horst et al., 2012), so there is a lack of research regarding the relationship between the early ERP components and the rotation angle of the object-based transformation with a stimulus of body image.

As far as the group difference was concerned, we found that when compared with non-athletes, the N2 amplitude of athletes was larger, and was significantly related to the performance of athletes’ perceptual and decision stages, but there was no group difference in N2 latency. The results partially supported Hypothesis 2. In other words, the athletes with a shorter RT at 0° also showed larger N2 amplitudes, which complements the results of our previous study (Feng et al., 2017). We believe that the athletes’ advantage in the perception and decision stages may be due to the athletes’ advantage in stimulus coding (perception stage) or movement speed (reaction stage). Consistent evidence was provided by a series of studies confirming the advantages of athletes’ simple RT and selective reaction (Kioumourtzoglou et al., 1998; Piras et al., 2014). Therefore, combined with the results of the present experiment, a larger N2 among the athletes showed that the athlete’s advantage is not only faster at perception and reaction in simple tasks, but that they could process and code the stimulation deeply and efficiently in high-level cognitive tasks (mental rotation). Lyu et al. (2017) compared the amplitude of N2 in hand mental rotation tasks between amputee patients and healthy individuals and found that the patients had a significantly larger N2 than healthy individuals did, which was related to the amputation years. Therefore, researchers believe that the N2 component in mental rotation represents the individual’s perception of stimulation (Lyu et al., 2017). In addition, some studies have found that athletes have advantages in the inhibition control and transfer effects of executive functions, which also supports the above explanation (Heppe et al., 2016). Compared with ordinary people, individuals with a weak ability of attentional modulation had smaller N2 amplitudes in the frontal area (Wascher et al., 2012). Athletes may show an increase in perceived efficiency due to their better attention adjustment ability. Studies using the UFOV (useful field of view) test to investigate the ability of visual-spatial attention confirmed that athletes had a better visual attention span and sustained attention ability, which could extract more effective information from the environment (Alves et al., 2013; Heppe et al., 2016).

In terms of RRN, the present study found that it appears in the parietal cortex and that the largest RRN changes are found at 550–750 ms in the OT condition and 400–600 ms in the ET condition, which is consistent with previous studies (Riečanský and Jagla, 2008; Horst et al., 2012; Lyu et al., 2017). According to the current study, RRN overlaps with P3 in a time window of 300–800 ms, forming a RRN (Wijers et al., 1989; Heil and Rolke, 2002). P3 activity is related to brain activity that is related to mental representation and may be closely related to the recognition and confirmation of stimuli (Donchin and Coles, 1988; Hayashi et al., 2005). Therefore, the increase in P3 amplitude indicates that the individual has recruited more cognitive resources during stimulation processing (Kok, 2001). With regards to RRN, it is considered to be closely related to the processing of mental rotation and represents the individual’s rotation operation of visual images (Rösler et al., 1990). Heil and Rolke (2002) found that the RRN component has nothing to do with the classification of stimuli and that the delay of the mental rotation process will cause the delay of RRN. Besides, previous studies demonstrated that the RRN amplitude of the mental rotation task can predict the individual’s RT (Riečanský and Jagla, 2008). In the present experiment, the angle effect of RRN showed a decreased trend at large angles in OT and ET conditions, which was consistent with the results of N2.

By investigating the RRN amplitude of mental rotation for athletes and non-athletes in the OT and ET conditions, we found that the RRN amplitude of athletes was significantly larger than that of non-athletes in both transformations. Moreover, this advantage was related to the performance of athletes in the rotation stage and their professional training years. This result verified Hypothesis 3 and was consistent with existing research. Yin (2015) investigated the mental rotation ability in different transformation types in expert and novice athletes. It was found that the expert group showed larger P3 amplitude only when they judged hand pictures (egocentric transformation), but not for the letters (object-based transformation). We used body images in OT conditions and revealed that athletes had better performance and larger amplitudes of RRN, so our results did not conflict with the research results of Yin. Additionally, researchers believe that the RRN or P3 component of mental rotation represents the correlation between task stimuli and subjects (Verleger and Śmigasiewicz, 2016). A study compared the mental rotation ability of amputees and healthy individuals and found that the RRN amplitude of amputees was significantly smaller than that of healthy individuals (Lyu et al., 2017). Therefore, due to their high-level skills and rich experience, athletes may conduct deeper processing of visual images related to their sport in mental rotation tasks. Extensive and efficient use of their cognitive resources causes the athletes to show larger RRN amplitudes. Moreover, a significant difference of the groups was found at 120° and 180° in ET conditions after which we analyzed the correlation between the RRN amplitude and the accuracy of athletes. It was found that the RRN amplitudes of P4 at 120° and P3 at 180° were positively correlated with their accuracy, which shows that the athletes’ advantage for large angles was reflected in not only their behavioral performance but also in their brain activity.

While the current study provides evidence for the time course of event-related brain potentials in athletes’ mental rotation with different spatial transformations, some limitations should be mentioned. First, Chinese divers often reach the international level at a younger age, so it is difficult to compare the mental rotation ability of divers with different sports levels (such as regional-level, college-level, and novice), so non-athletes were selected as the control group in the present study. Additionally, only a paper-plane rotation axis was used in the experiment. However, many sports use multi-axis rotation including the vertical or horizontal axis of the body. Based on this, future studies are needed to compare divers with different expertise levels and to test the mental rotation ability of different rotation axes of the body.

## Conclusion

This study found that the athletes’ N2 amplitudes were larger at 0°, 60°, and 120° in the ET condition and only at 0° in the OT condition. The RRN amplitudes of the athletes were larger at 0°, 60°, and 120° in the OT condition and at all angles in the ET condition. These two advantages were related to the athletes’ stage performance and training years, respectively. In mental rotation tasks with body images, athletes and non-athletes have differences not only in behavior but also in ERP effects.

## Data Availability Statement

The original contributions presented in the study are included in the article/Supplementary Material, further inquiries can be directed to the corresponding author.

## Ethics Statement

The studies involving human participants were reviewed and approved by the Ethics Committee of the Shanghai University of Sport (2017036). The patients/participants provided their written informed consent to participate in this study.

## Author Contributions

TF wrote the original draft, acquired funding, analyzed the data, conducted the methodology and formal analysis, and contributed to writing—review and editing. YL collected the data, revised the manuscript, and supervised all phases of the work. All authors contributed to the article and approved the submitted version.

## Conflict of Interest

The authors declare that the research was conducted in the absence of any commercial or financial relationships that could be construed as a potential conflict of interest.
